# Naproxcinod shows significant advantages over naproxen in the *mdx* model of Duchenne Muscular Dystrophy

**DOI:** 10.1186/s13023-015-0311-0

**Published:** 2015-08-22

**Authors:** Daniela Miglietta, Clara De Palma, Clara Sciorati, Barbara Vergani, Viviana Pisa, Antonello Villa, Ennio Ongini, Emilio Clementi

**Affiliations:** Nicox Research Institute, 20091 Bresso, Milano, Italy; Unit of Clinical Pharmacology, Department of Biomedical and Clinical Sciences, Consiglio Nazionale delle Ricerche Institute of Neuroscience, University Hospital “Luigi Sacco”, University of Milan, 20157 Milan, Italy; Division of Regenerative Medicine, Stem Cells & Gene Therapy, San Raffaele Scientific Institute, 20132 Milan, Italy; Consorzio MIA (Microscopy and Image Analysis), University of Milano-Bicocca, 20900 Monza, Italy; Scientific Institute, IRCCS E. Medea, 23842 Bosisio Parini, Lecco, Italy

**Keywords:** Naproxcinod, Nitric oxide, Duchenne muscular dystrophy, Inflammation, Fibrosis, *mdx* mouse model

## Abstract

**Background:**

In dystrophin-deficient muscles of Duchenne Muscular Dystrophy (DMD) patients and the *mdx* mouse model, nitric oxide (NO) signalling is impaired. Previous studies have shown that NO-donating drugs are beneficial in dystrophic mouse models. Recently, a long-term treatment (9 months) of *mdx* mice with naproxcinod, an NO-donating naproxen, has shown a significant improvement of the dystrophic phenotype with beneficial effects present throughout the disease progression. It remains however to be clearly dissected out which specific effects are due to the NO component compared with the anti-inflammatory activity associated with naproxen. Understanding the contribution of NO *vs* the anti-inflammatory effect is important, in view of the potential therapeutic perspective, and this is the final aim of this study.

**Methods:**

Five-week-old *mdx* mice received either naproxcinod (30 mg/kg) or the equimolar dose of naproxen (20 mg/kg) in the diet for 6 months. Control *mdx* mice were used as reference. Treatments (or vehicle for control groups) were administered daily in the diet. For the first 3 months the study was performed in sedentary animals, then all mice were subjected to exercise until the sixth month. Skeletal muscle force was assessed by measuring whole body tension in sedentary animals as well as in exercised mice and resistance to fatigue was measured after 3 months of running exercise. At the end of 6 months of treatment, animals were sacrificed for histological analysis and measurement of naproxen levels in blood and skeletal muscle.

**Results:**

Naproxcinod significantly ameliorated skeletal muscle force and resistance to fatigue in sedentary as well as in exercised mice, reduced inflammatory infiltrates and fibrosis deposition in both cardiac and diaphragm muscles. Conversely, the equimolar dose of naproxen showed no effects on fibrosis and improved muscle function only in sedentary mice, while the beneficial effects in exercised mice were lost demonstrating a limited and short-term effect.

**Conclusion:**

In conclusion, this study shows that NO donation may have an important role, in addition to anti-inflammatory activity, in slowing down the progression of the disease in the *mdx* mouse model therefore positioning naproxcinod as a promising candidate for treatment of DMD.

**Electronic supplementary material:**

The online version of this article (doi:10.1186/s13023-015-0311-0) contains supplementary material, which is available to authorized users.

## Background

Duchenne Muscular Dystrophy (DMD) is the most common form of muscular dystrophy, affecting approximately one in every 3,500 live male births. It is caused by mutations in the dystrophin gene [1]. DMD patients exhibit progressive skeletal muscle degeneration and weakness as well as cardiomyopathy [2]. Dystrophin-deficient muscle exhibits chronic inflammation, and over time, muscle fibres are steadily replaced with fibrotic and fatty tissue [3]. Effective treatment for DMD is lacking, resulting in premature death often before the age of 30 due to respiratory muscle weakness and/or cardiomyopathy [4]. Currently, corticosteroids constitute the primary treatment option for muscle dysfunction in DMD. However, despite the extension of ambulation by 2–3 years and mitigation of pulmonary complications, the use of steroids is associated with serious side effects [4].

Dystrophin deficiency in muscles results in the loss of a large transmembrane protein complex, the dystrophin-glycoprotein complex (DGC), which plays a structural role in maintaining sarcolemmal integrity [5]. Among the DGC proteins is the muscle-specific splice variant of neuronal nitric oxide synthase μ (nNOSμ), which catalyses the synthesis of nitric oxide (NO) in the skeletal muscle. Therefore, the loss of dystrophin causes a secondary deficiency of nNOSμ, which is demonstrated to significantly contribute to the pathogenesis and progression of DMD [6, 7]. NO is an important regulatory signal for a large number of physiological effects in the muscle that are fundamental for muscle integrity and function [8]. The mislocalisation and reduction of nNOS expression and consequent reduction in NO generation have been associated with impaired skeletal muscle contraction, vascular dilation, and eventual muscle damage [9], as well as impaired muscle regeneration [10, 11]. It has been shown that NO supplementation improves blood flow and oxygen supply to contracting muscle, thus reducing muscle ischemia and increasing glucose uptake, muscle contraction and resistance to fatigue [12, 13]. NO has also been documented to mediate activation of satellite precursor cells, providing new donor cells for skeletal muscle growth and muscle repair from injury or disease [10, 11]. Thus, increasing NO in muscle can promote regeneration of dystrophic muscles.

A variety of pharmacological and genetic approaches aimed at regulating NO supply to the muscle have been shown to slow disease progression in several animal models of skeletal muscular dystrophies, i.e. the *mdx* and α-sarcoglycan null mice. Specifically, overexpression of nNOS or treatment with NO donors such as molsidomine and isosorbide dinitrate (ISDN) attenuate skeletal muscle inflammation and necrosis and/or improve exercise performance in different mouse models of muscular dystrophy [14–19].

A new class of NO-donating drugs called cyclooxygenase (COX)-inhibiting NO donors (CINODs) has shown beneficial effects in preclinical models of muscular dystrophies. This class of molecules combines a classic COX inhibitor with a NO-donating moiety to produce a dual pharmacological action [20]. Chronic treatment (6–12 months) of *mdx* and α-sarcoglycan null mice with the CINOD HCT 1026, an NO-donating flurbiprofen, has been shown to markedly improve muscle morphology and reduce muscle necrosis, inflammation, and fatigue [21]. Similar effects have been observed in α-sarcoglycan null mice treated with NCX 320, an NO-donating ibuprofen [22]. HCT 1026 also reverses functional muscle ischemia in *mdx* mice, an effect which is maintained following prolonged treatment, negating the concern that tolerance to the drug might develop with chronic use [12]. This is a potential drawback for some commonly-used NO donors such as the organic nitrates (e.g., nitroglycerin and ISDN), since their vasodilating properties decline following continuous exposure [23]. Nitrate tolerance has also been reported in the regulation of skeletal muscle blood flow [24], also suggesting a potential limitation of this class of compounds for chronic treatment of muscle disorders. Another important side effect associated with organic nitrates is rapid hypotension due to the fast release of a large amount of NO [25]. This effect has not been reported with CINODs since they release NO at low concentrations for prolonged periods [20]. Therefore, this new class of drugs is effective at improving the dystrophic phenotype without the limitations of nitrate tolerance and hypotension associated with the commonly-used organic nitrates.

Recently, the most advanced compound belonging to the CINOD class, naproxcinod, a NO-releasing naproxen, has been demonstrated to improve the skeletal and cardiac disease phenotype in *mdx* model of muscular dystrophy after long-term treatment. In that study, in which a wild-type group was used in order to establish the recovery score obtained with the drug, treated animals showed improved skeletal muscle and cardiac function, reduced muscle inflammation and cardiac fibrosis and improved skeletal muscle blood flow [26]. Naproxcinod, has been widely investigated in animal and clinical studies, including Phase III clinical trials for osteoarthritis in more than 2,700 adult patients; the safety database includes more than 4,000 patients [27]. Therefore, naproxcinod has been considered the ideal candidate among CINODs to be developed for the treatment of DMD.

Although naproxcinod has been shown to be effective in the *mdx* mouse model of DMD, it is unclear to what extent the NO properties contribute to its effects beyond those related to naproxen-dependent anti-inflammatory activity. Therefore, this study was designed to evaluate the NO-related activity of naproxcinod by comparison with the anti-inflammatory naproxen in the *mdx* mouse model following 6 months of treatment and evaluating specifically the extent of benefit [28] as valid parameter of drug efficacy.

## Methods

### Mice and treatment

Male *mdx* (C57BL/10-*mdx*) mice, 5 weeks of age, were obtained from Jackson Laboratories (Bar Harbor, Maine) and were handled according to Italian law for care and use of laboratory animals (D.L. 26/2014), as well as European Directive (2010/63/UE). The experimental procedures used respected the standard operating procedures for pre-clinical tests in *mdx* mice available on http://www.treat-nmd.eu/research/preclinical/dmd-sops/.

A study by Uaesoontrachoon and coworkers [26] demonstrated that 21 mg/kg/day of naproxcinod could be considered an effective dose in *mdx* mice, while an higher dose of 41 mg/kg/day lost beneficial activity. Thus, a further study was designed to test an intermediate concentration of naproxcinod of 30 mg/kg/day in term of efficacy in the DMD mouse model. In particular, two doses of naproxcinod (10 and 30 mg/kg) were given to *mdx* mice (*n* = 10 per group) for 7 months starting at 5 weeks of age. The compound was administered daily in the diet (Mucedola, Milano, Italy), and the same diet without drug was given to control *mdx* mice (*n* = 10). Following 4 and 7 months of treatment, resistance to fatigue was assessed by running treadmill assay. At the end of treatment, morphological analysis of tibialis anterior was performed.

Based on the results obtained in this exploratory study, the dose of 30 mg/kg was identified as a further effective dose of naproxcinod. According to this, five-week old *mdx* mice (10 mice/group) were treated orally with either naproxcinod (30 mg/kg), or an equimolar dose of naproxen (20 mg/kg), starting at 1 month of age for 6 months. Control *mdx* mice were used as reference. Treatments (or vehicle for control groups) were administered daily in the diet. Body weight and food intake were monitored weekly for 5 months. For the first 3 months the study was performed in sedentary animals and skeletal muscle force was assessed every month. Then all mice were subjected to exercise for additional 3 months. Finally, resistance to fatigue and skeletal muscle force were measured after 3 months of running exercise (i.e. 6 months of treatment) to evaluate the effects of treatment on muscle function in exercised animals. Once completed the functional tests, animals were sacrificed for histological analysis and measurement of blood and skeletal muscle naproxen levels. Results are reported as the extent of benefit between the treated and untreated groups of *mdx* mice [28].

### Treadmill to impact on *mdx* phenotype

In order to impact on *mdx* phenotype, in the second study all mice, from the third month of treatment, were subjected to a 30 min run on a horizontal treadmill using the Exer 3/6 Treadmill (Columbus Instruments, USA) at 10 m/min, twice a week as described in the TREAT-NMD SOP DMD_M.2.1.001 [29]

### Treadmill to assess dystrophic state

Resistance to fatigue was assessed by treadmill running to exhaustion, using the Exer 3/6 Treadmill; (Columbus Instruments, Columbus, OH) and modelling the six minute walk test currently recommended as the key outcome measure in human trials for DMD. The exhaustion treadmill test was performed after an appropriate training period and four tests were performed on the same animal, allowing one week between each test.

In the first study, the exercise resistance test was performed for four weeks (once a week) at an uphill inclination of 30 %, for 5 min at 1 m/min modifying protocols already described in literature [17, 30, 31]. Then, the speed was increased by 1 m/min every 2 min until exhaustion.

In the second study, the assay consisted of horizontal running for 5 min at 5 m/min, and then the speed was increased by 1 m/min each minute until exhaustion as reported in the TREAT-NMD SOP (DMD M.2.1.003). The test measured time of running and total distance run by each mouse until exhaustion.

### Skeletal muscle force: Whole body tension assay

*In vivo* skeletal muscle force was measured every month for the first 3 months of treatment by whole body tension (WBT) in sedentary *mdx* mice, using Grass FT03 transducer and following the specific SOP (DMD M.2.2.006). The WBT procedure is used to determine the ability of mice to exert tension in a forward pulling manoeuvre that is elicited by stroking the tail. It is thought to reflect the maximal acute phasic force the mouse can achieve to escape a potentially harmful event. The total phasic (or acute) forward pulling tension (FPT) exerted by the fore- and hind limb musculature of mice were recorded and normalized by body weight. *In vivo* muscle force was also measured at 6 months of treatment following the 4 weeks of treadmill exhaustion test, to assess the impact of exercise on muscle function. The results were expressed as WBT5 and WBT10, which are calculated as the top 5 and 10 FPTs, respectively, divided by body weight.

### Histology

At the end of treatment, muscle samples (from diaphragm and tibialis anterior muscles) were frozen in liquid nitrogen-cooled isopentane and serial 10 μm thick sections were cut with a Leica cryostat. Sections were then stained with haematoxilin and eosin (H&E) according to the standard procedure. At least 3–4 random images for each muscle were taken at 10x magnification with a DMI 4000B microscope (Leica Microscopy Systems, Heerbrugg, Switzerland). Image acquisition was performed by Leica LAS AF software version 2.5.0.6735 and analysed in a blinded fashion using digitised imaging systems (Image J - National Institute of Health) to evaluate infiltrated inflammatory areas.

In the second study, skeletal muscle fibrosis was also measured by the Masson’s Trichrome staining for detecting collagen inside the muscle, according to the standard protocol [22]. The fibrotic area corresponding to the area stained in blue was quantified and compared to the total area of the tissue section through image analysis software (Image J - NIH).

In addition to skeletal muscles, in the second study, heart and gastric samples were also removed, fixed in 10 % formalin and sectioned. Both stomach and heart were sectioned and stained for H&E by Consorzio MIA (Monza, Italy). Five random digital images of each gastric sample were taken using an Eclipse E600 (Nikon, Japan) microscope. Picro-Sirius red staining was performed to measure the degree of cardiac fibrosis according to the standard procedure [26]. Eight random Picro-Sirius stained sections of each heart sample were digitalized using the Aperio ScanScop XT System (Aperio Technologies; Vista, CA), and blinded analysis was done using Image J (NIH), with additional threshold colour plug-ins to process images. Pixels corresponding to the area stained in red were normalised to the total pixel area of the tissue image and the results expressed as percentage of fibrosis.

### Naproxen blood level quantification

At the end of the second study, animals were sacrificed and blood samples were taken by heart puncture. 50 μl of each blood sample were protein-precipitated by adding 150 μl of acetonitrile (ACN) and 10 μl of dimethyl sulfoxide (DMSO) in triplicate, vortex-mixed and centrifuged for 10 min at 4 °C (3200 g); the supernatant was transferred to a clean tube and kept at −80 °C until LC-MS/MS analysis of naproxen.

The analytical system comprised a Sciex API 4000 mass spectrometer (Apply Byosistem, Foster City, CA), a CTC HTS PAL autosampler (LEAP Technologies, Carrboro, NC), and Agilent LC-1200 pump (Agilent Technologies, Santa Clara, CA). The samples were analyzed using reverse-phase chromatography (Poroshell 120 EC-C18 2.1x50mm 2.7 μm; Agilent Technologies, Santa Clara, CA) and the column temperature was maintained at 40 °C. A 1.7-min linear gradient from 70 to 0 % of mobile phase A (formic acid 0.1 %) was used at the flow rate of 0.5 ml/min, and mobile phase B was acetonitrile containing 0.1 % of formic acid. Positive ion multiple reaction monitoring with parent /fragment 231.15 → 185.05 was used to monitor naproxen levels in the experiment.

### Naproxen skeletal muscle level quantification

At the end of treatment, animals were sacrificed and gastrocnemius samples were removed, immediately frozen in liquid nitrogen and stored at −80 °C until analyzed. Then, each sample was homogenized in a mortar in the presence of liquid nitrogen and three volumes of ACN were added. Thereafter, samples were vortex-mixed and centrifuged for 10 min at 4 °C (3200 g). The supernatant was transferred to a clean tube and analyzed by LC-PDA for naproxen level quantification. Data were reported as ng of naproxen in mg of tissue.

Liquid chromatography was performed on ACQUITY UPLC system (Waters Corp., Milford, MA) with autosampler and column oven enabling temperature control of analytical column. AQUITY UPLC BEH C18 column (2.1x50mm, 1.7 μm; Waters Corp., Milford, MA) was employed. The column temperature was maintained at 40 °C. A 1.7-min linear gradient from 60 to 0 % of mobile phase A (formic acid 0.1 %) was used at the flow rate of 0.5 ml/min, and mobile phase B was methanol containing 0.1 % of formic acid.

PDA detection was carried out on ACQUITY UPLC PDA detector (Waters Corp., Milford, MA). Wavelength of 230 nm was used to monitor naproxen levels in the experiment.

### Statistical analysis

Results were expressed as the means ± SEM. The differences between mean values were assessed by one-way ANOVA, followed by the Tukey post-hoc test or by two-way ANOVA followed by the Bonferroni post-hoc test when appropriate. A P-value < 0.05 was considered statistically significant.

## Results

### Naproxcinod at 30 mg/kg is effective in the mdx mouse model of DMD

**Fig. 1:**
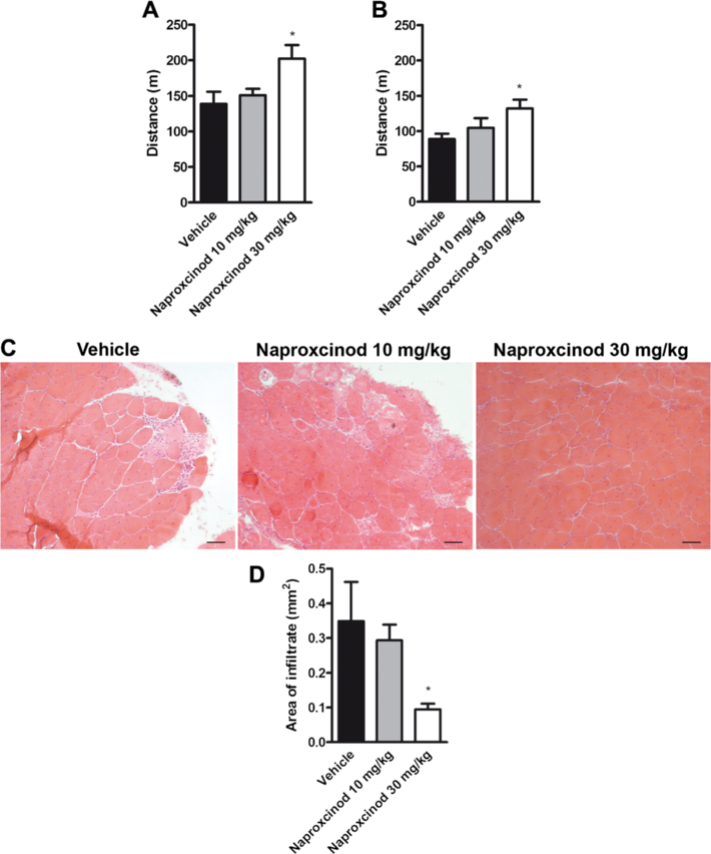
30 mg/kg of naproxcinod is an effective dose in *mdx* mice. In a first exploratory study, *mdx* mice were treated with two doses of naproxcinod (10 and 30 mg/kg) for 7 months. Locomotor function, assessed by treadmill running to exhaustion, was measured after 4 (**a**) and 7 months (**b**) of treatment. Quantification of inflammation in the tibialis anterior muscle of *mdx* mice treated with vehicle, 10 or 30 mg/kg naproxcinod was performed at the end of treatment. **c** Representative histological images of the tibialis anterior muscle after H&E staining and **d** quantification of inflammatory infiltrate area. Data are presented as mean ± SEM. *represents the comparison between vehicle and all the treatment groups. One-way ANOVA followed by Tukey post-hoc test. *P < 0.05. *N* = 8-10 mice/group. Bar = 100 μm

Two doses of naproxcinod (10 and 30 mg/kg) were tested in the *mdx* mouse model to better assess drug efficacy and range of activity. *Mdx* mice were treated for 7 months with vehicle and a low or high dose of naproxcinod incorporated into the diet starting at 5 weeks of age. The dose of 30 mg/kg of naproxcinod showed a significant improvement (*P* < 0.05) in running distance until exhaustion compared to vehicle-treated *mdx* mice, with the distance run 46 % and 49 % greater at 4 and 7 months respectively (Fig. 1a and b). In addition, tibialis anterior muscles from *mdx* mice receiving 30 mg/kg naproxcinod for 7 months showed a significantly smaller area of infiltrate ( −70 %, *P* < 0.05) than *mdx* mice treated with vehicle (Fig. 1c and d). Conversely, the lower dose of 10 mg/kg confirmed a slight effect on muscle function which did not reach statistical significance (Fig. 1a and b), and no effects on muscle structure (Fig. 1c and d), as already published [26]. Based on these results, the dose of 30 mg/kg was identified as a further effective dose and selected for the study in comparison with naproxen.

### Naproxcinod- and naproxen-treated mice have similar body weights and food intake

*Mdx* mice were given either naproxcinod (30 mg/kg) or an equimolar dose of naproxen (20 mg/kg) incorporated into the diet starting at 5 weeks of age. The same diet without any drug was used as the control diet. All animals were weighed every week for 5 months. There were no statistically significant differences in body weight between the vehicle group and either treated group (Additional file 1a). Food was weighed every week for 5 months and no significant difference in food intake/mouse/day was detected between the groups (Additional file 1b).

### Naproxcinod improved skeletal muscle force in sedentary mdx mice

*In vivo* skeletal muscle force was measured every month for the first 3 months of treatment by whole body tension (WBT). Following the first month of treatment, both naproxen and naproxcinod significantly improved skeletal muscle force as indicated by an increase in the parameters WBT5 (55 % for naproxen, 80 % for naproxcinod, *P* < 0.01 *vs* vehicle) and WBT10 (51 % for naproxen, 76 % for naproxcinod, *P* < 0.01 *vs* vehicle; Additional file 2a and b). Interestingly, for WBT10, naproxcinod showed a significantly greater improvement of muscle force compared to naproxen (16 % greater, *P* < 0.05).

The significant beneficial effects of both drugs on skeletal muscle force assessed by WBT were maintained with treatment over the following 2 months (WBT5: 44 % for naproxen, 46 % for naproxcinod, *P* < 0.001 *vs* vehicle; WBT10: 48 % for naproxen, 56 % for naproxcinod, *P* < 0.001 *vs* vehicle, Additional file 2c and d) and 3 months (WBT5: 50 % for naproxen, 56 % for naproxcinod, *P* < 0.001 *vs* vehicle; WBT10: 55 % for naproxen, 63 % for naproxcinod, *P* < 0.001 *vs* vehicle; Additional file 2e and f). However, there was no significant difference between the two drug treatments.

### Naproxcinod increased resistance to fatigue in exercised mdx mice compared to naproxen

**Fig. 2:**
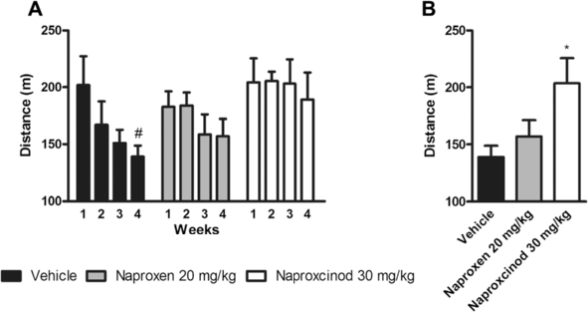
Naproxcinod improves resistance to fatigue in exercised *mdx* mice. Resistance to fatigue was assessed by treadmill running to exhaustion following 6 months of treatment in exercised *mdx* mice with either vehicle (black bar), 20 mg/kg naproxen (grey bar) or 30 mg/kg naproxcinod (white bar). **a** Measurements made once a week for four consecutive weeks and **b** data obtained during the fourth week of running to exhaustion. Data are presented as mean ± SEM. # represents the comparison between each time point. Two-way ANOVA followed by Bonferroni post-hoc test. *represents the comparison between vehicle and all the treatment groups. One-way ANOVA followed by Tukey post-hoc test. * and # P < 0.05. N = 8-10 mice/group

Resistance to fatigue was assessed by the treadmill running assay at the 6-month time point following 3 months of running exercise twice/week (30 min at 10 m/min speed). Exercised vehicle-treated *mdx* mice showed a dramatic increase in fatigability between the start and the fourth week of running to exhaustion (*P* < 0.05; Fig. 2a). Naproxen-treated *mdx* mice also showed a trend of increased fatigability throughout the 4 weeks of running. However, naproxcinod-treated mice showed the same resistance to fatigue as at the start of the running session (Fig. 2a). For running performance at the fourth week, 30 mg/kg naproxcinod significantly improved resistance to fatigue with a 46 % mean increase (P < 0.05) in distance travelled compared to vehicle. In contrast, naproxen showed only a trend toward improvement of about 10 % (Fig. 2b). Moreover, naproxcinod resulted in better protection against fatigue compared to naproxen (by about 30 %), although this effect did not reach statistical significance because of inter-animal variability.

### Naproxcinod improved skeletal muscle force in exercised mdx mice compared to naproxen

**Fig. 3:**
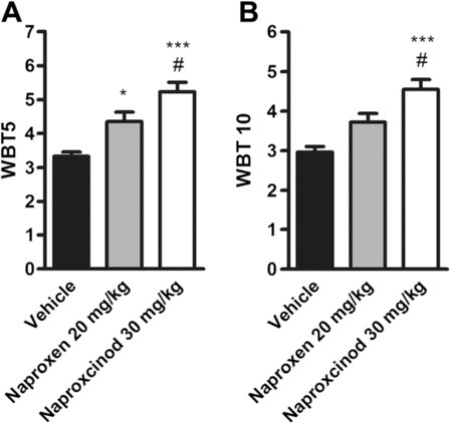
Naproxcinod improves skeletal muscle force in exercised *mdx* mice. **a** WBT5 and **b** WBT10 measured following 6 months of treatment in exercised *mdx* mice treated with vehicle (black bar), 20 mg/kg naproxen (grey bar), or 30 mg/kg naproxcinod (white bar). Data are presented as mean ± SEM. *represents the comparison between vehicle and treatment groups. # represents the comparison versus the naproxen-treated group. One-way ANOVA followed by Tukey post-hoc test. * and # *P* < 0.05, ***P < 0.001. *N* = 8-10 mice/group

*In vivo* skeletal muscle force was assessed by WBT assay at 6 months of treatment in exercised *mdx* mice, 24 h after the 4 weeks of running to exhaustion. Naproxcinod treatment led to a significant improvement of both WBT5 and WBT10 (57 % and 53 % respectively, *P* < 0.001) compared to vehicle-treated *mdx* mice (Fig. 3a and b). In addition, the WBT values at 6 months of treatment in exercised mice were similar to those after 2 and 3 months of treatment in sedentary *mdx* mice, thus suggesting that naproxcinod maintains its efficacy up to 6 months of treatment and protects against exercise-induced skeletal muscle weakness. Conversely, naproxen, which at 2 and 3 months of treatment in sedentary conditions showed an effect in the WBT assay similar to that of naproxcinod, did not maintain its efficacy when tested at 6 months in trained *mdx* mice. In particular, naproxen-treated *mdx* mice showed only 30 % improvement for WBT5 (*P* < 0.05) and 25 % for WBT10 (NS) compared to vehicle-treated *mdx* mice. Therefore, the beneficial effects of naproxcinod on skeletal muscle force were significantly greater (around 20 % for both WBT5 and WBT10, *P* < 0.05) than those of naproxen, thus suggesting an important role for NO in counteracting exercise-induced skeletal muscle weakness.

### Naproxcinod reduced diaphragm inflammation and fibrosis in mdx mice

**Fig. 4:**
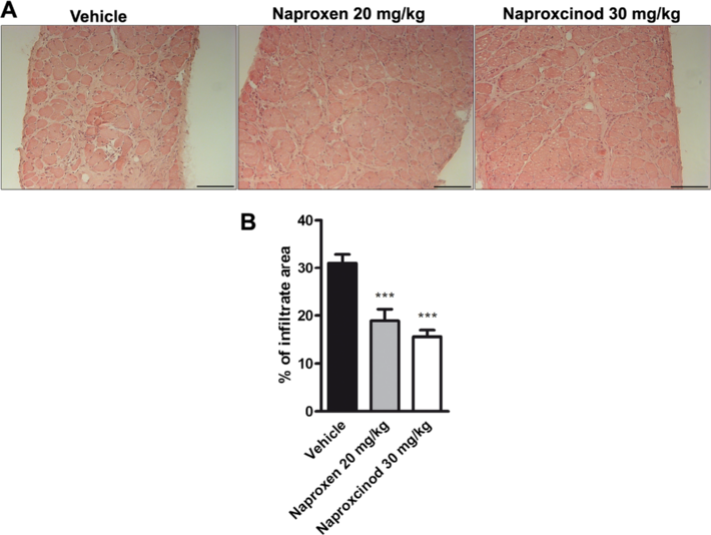
Naproxcinod significantly reduces inflammation in diaphragm of *mdx* mice. Quantification of inflammation in the diaphragm of *mdx* mice treated with vehicle (black bar), 30 mg/kg naproxcinod (white bar) or 20 mg/kg naproxen (grey bar) following 6 months of treatment. **a** Representative histological images of the diaphragm muscle after H&E staining and **b** quantification of the area of inflammatory infiltrates, expressed as a percentage of muscle cross section. Data are presented as mean ± SEM. *represents the comparison between vehicle and treatment groups. One-way ANOVA followed by Tukey post hoc test. ***P < 0.001. *N* = 4-5 mice/group. Bar = 100 μm

**Fig. 5:**
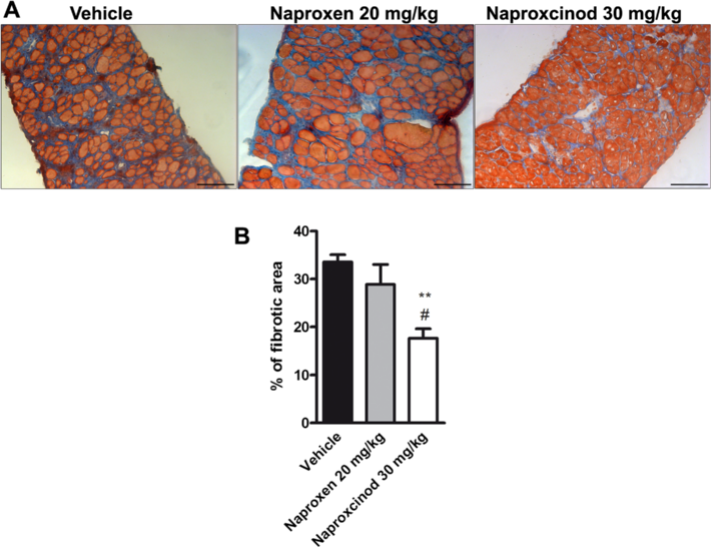
Naproxcinod significantly reduces fibrosis in diaphragm of *mdx* mice. Quantification of fibrosis content assessed by Masson’s Trichrome staining in the diaphragm of *mdx* mice treated with vehicle (black bar), 30 mg/kg naproxcinod (white bar), or 20 mg/kg naproxen (grey bar) following 6 months of treatment. **a** Representative histological images of the diaphragm and **b** quantification of fibrotic area, expressed as a percentage of muscle cross section. Data are presented as mean ± SEM. *represents the comparison between vehicle and treatment groups. #represents the comparison versus the naproxen-treated group. One-way ANOVA followed by Tukey post-hoc test. ** *P* < 0.01; # *P* < 0.05. N = 4-5 mice/group. Bar = 100 μm

Histology of H&E-stained sections of the diaphragm muscles was assessed for inflammation. Histology profile of *mdx* diaphragm muscles shows typical dystrophic features, such as the alteration of the muscle architecture with areas of infiltrates and a large non-muscle area, likely due to the deposition of fibrotic and adipose tissue (Fig. 4a). A blinded morphometric analysis revealed a significant reduction in inflammatory infiltrate when mice were treated with naproxen ( −39 %, *P* < 0.001) and naproxcinod (−50 %, *P* < 0.001), respectively (Fig. 4a and b). Masson Trichrome staining for collagen revealed, as expected, fibrosis in the diaphragm sections of *mdx* mice. Naproxen-treated mice showed the same level of fibrosis observed in vehicle-treated *mdx* mice, while treatment with naproxcinod significantly reduced diaphragm fibrosis deposition compared to both vehicle ( −47 %, *P* < 0.01) and naproxen ( −39 %, *P* < 0.05) (Fig. 5a and b).

### Naproxcinod reduced cardiac fibrosis in mdx mice

**Fig. 6:**
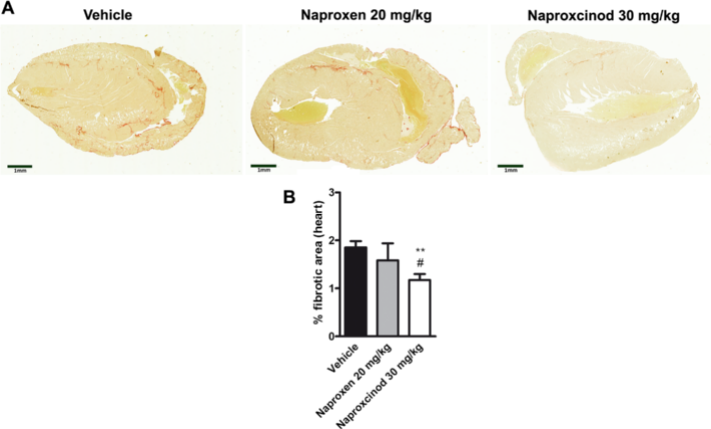
Naproxcinod significantly reduces cardiac fibrosis. Quantification of the cardiac fibrosis assessed by Picro-Sirius staining in *mdx* mice treated with vehicle (black bar), 30 mg/kg naproxcinod (white bar), or 20 mg/kg naproxen (grey bar) following 6 months of treatment. **a** Representative images of the heart and **b** quantification of the area of fibrosis in the heart, expressed as a percentage of the whole heart. Data are presented as mean ± SEM. *represents the comparison between vehicle and the treatment groups. #represents the comparison versus the naproxen-treated group. One-way ANOVA followed by Tukey post-hoc test. **P < 0.01; # *P* < 0.05. *N* = 5-10 mice/group. Bar = 1 mm

Given the effects on fibrosis with naproxcinod in diaphragm samples, we also assessed the level of fibrosis in the heart using Picro-Sirius red staining. Heart samples from *mdx* mice showed fibrosis deposition as previously reported [32, 26]. As in the diaphragm muscle, treatment with 30 mg/kg naproxcinod caused a significant reduction in cardiac fibrosis compared to either vehicle or naproxen treated *mdx* mice ( −35 %, *P* < 0.01 and - 29 %, *P* < 0.05, respectively), while the equimolar dose of naproxen did not show any significant effect (Fig. 6a and b).

### Naproxcinod caused less gastric damage than naproxen in mdx mice

Since non-steroidal anti-inflammatory drugs are known to induce gastric mucosal damage following chronic treatment, a blinded qualitative morphological analysis of the stomach of these animals was performed. As expected, a 6-month treatment with 20 mg/kg naproxen altered the gastric mucosa compared to vehicle-treated *mdx* mice, markedly reducing the mucosal layer (especially foveolar cells). In contrast, naproxcinod caused a clearly reduced level of gastric damage (Additional file 3).

### Naproxcinod showed reduced bioavailability compared to naproxen

In order to verify if naproxcinod given at a dose equimolar to naproxen was able to release the same amount of naproxen following oral administration, we examined the blood levels of drug of treated mice. Following 6 months of treatment with either naproxcinod (30 mg/kg) or the equimolar dose of naproxen (20 mg/kg) incorporated into the diet, the blood levels at steady state of naproxen were measured by LC-MS/MS. Blood levels of naproxen were 12.4 ± 2.6 μM, while those in blood samples of animals treated with naproxcinod were 4.1 ± 1.3 μM. These data underline a reduced bioavailability of naproxcinod compared to naproxen of about 3-fold. Similar results were found in gastrocnemius muscle samples, where animals treated with naproxcinod showed concentrations of 0.07 ± 0.01 ng/mg of tissue, while those treated with naproxen showed 0.2 ± 0.1 ng/mg of tissue.

## Discussion

Naproxcinod is the most advanced of a new class of anti-inflammatory agents, CINODs, in which a standard COX-inhibiting NSAID such as naproxen is linked to a NO-donating moiety to produce dual pharmacological actions [33, 20]. Naproxcinod, upon absorption, is metabolized to naproxen and the NO–donating moiety, which in turn releases NO through enzyme bioactivation.

This class of drugs was initially developed as therapeutic alternatives to the NSAIDs for the treatment of osteoarthritis (OA). The addition of the NO-donating moiety was aimed at reducing common adverse effects of chronic NSAID use, such as gastrointestinal damage and increased blood pressure. Indeed, naproxcinod was shown in several clinical trials to be effective in relieving the signs and symptoms of OA compared to placebo [27, 34–36], but with a lower incidence of hypertension compared to NSAIDs [37, 38]. Moreover, it has been demonstrated that the effects of naproxcinod ascribed to NO such as the control of blood pressure (BP) persist over time [38] up to 13 weeks, suggesting that the compound does not lead to development of nitrate tolerance with the chronic use.

However, FDA approval was not granted, as additional long-term clinical studies were requested to differentiate the drug from reference NSAIDs. Recent preclinical studies suggest, however, that CINODs are effective in muscular dystrophy models (21).

The beneficial effects of CINOD treatment in dystrophic mice are related to a combination of effects ranging from reduced inflammation and necrosis and preserved regenerative capacity of muscle to improved skeletal muscle blood flow. A previous study with naproxcinod demonstrated that it was effective at improving the dystrophic phenotype in the *mdx* mouse model following long-term treatment [26]. Specifically, naproxcinod improved skeletal muscle and cardiac functions, and reduced skeletal muscle inflammation as well as cardiac fibrosis following 9 months of treatment. Importantly, these beneficial effects persisted throughout disease progression, without adverse side effects such as those observed with prednisolone, the current treatment option for DMD. Therefore, the compound was shown to be effective and better tolerated than the therapy currently used for DMD. However, the studies performed to date had not yet demonstrated the specific contribution of the NO-donating moiety over the COX-inhibiting activity. Therefore, in the present work, naproxcinod was studied in comparison with its parent drug naproxen, to investigate any additional effects of NO-donation over and above COX-inhibition.

The dose of 30 mg/kg used in this study was selected in order to optimize drug dosage, i.e. to identify the maximal effective dose, higher than the one previously found effective, but below the 41 mg/kg, a dose at which naproxcinod lost its efficacy in *mdx* mice [26]. Although no data are available explaining the loss of efficacy observed with the high dose of naproxcinod, the involvement of nitrosylation in causing this effect is a possibility. *S*-nitrosylation of the ryanodine receptor (RyR1) indeed contributes to promote muscle weakness in *mdx* skeletal muscles by altering Ca^2+^ homeostasis [39], and S-nitrosylation of SIRT1 (silent mating type information regulation 2 homolog) results in the induction of pro-inflammatory pathways [40]. However, here we did not observe any muscle weakness. Interestingly, it is not unusual to observe bell-shaped dose response curves with drugs used for the treatment of DMD condition (i.e. ataluren) [41]. This suggests that caution in dose selection is particularly relevant in a such clinical setting as DMD.

Based on the results obtained, the dose of 30 mg/kg was fully effective at improving skeletal muscle function and morphology. In addition, the dose chosen is in the upper range of a clinically daily effective dose (i.e. 750 mg bid which corresponds to 20–25 mg/kg/day). In contrast, 10 mg/kg did not induce any significant beneficial effects on the parameters measured. Therefore, the dose of 30 mg/kg was used for the full study in comparison with naproxen.

Treatment of *mdx* mice with naproxcinod (30 mg/kg) for 6 months did not result in any effects on body weight or food consumption compared to both control and naproxen-treated animals, suggesting that the compound was safe throughout the duration of treatment. Naproxcinod treatment significantly improved skeletal muscle force already after the first month of treatment in sedentary animals; this effect was also maintained throughout the duration of treatment even when mice were forced to exercise. The equimolar dose of naproxen (20 mg/kg) caused an improvement of muscle force that was comparable to naproxcinod only at 2 and 3 months of treatment in sedentary animals.

However, when animals were forced to run, a condition in which muscle damage is enhanced [42–44], naproxen’s effects on muscle force were significantly lower than those of naproxcinod. Similar effects were also observed on locomotor function. When exercised mice were forced to run until exhaustion, only those treated with naproxcinod showed a significantly improved resistance to fatigue compared to control animals. These data on muscle function could suggest that in sedentary adult *mdx* mice, inflammation is likely the main pathologic event, and therefore the reduction of inflammation is sufficient to improve muscle function. When the severity of the phenotype is increased by forcing animals to exercise, achieving beneficial effects by these drugs requires additional mechanisms beyond anti-inflammatory action. Indeed, under such conditions, the assumption is that NO, released by naproxcinod, could become relevant over the anti-inflammatory action.

In particular, the pathophysiology of DMD also includes the exhaustion of the myogenic pool of cells and necrosis together with fibrosis and fat deposition, a condition in which the role of NO has been shown to be important [45, 11]. Indeed, NO is known to play an important role in stimulating skeletal muscle regeneration, thus maintaining functional muscle tissue for longer [10, 46]. The improvement in resistance to fatigue may also be explained by the ability of NO donors to alleviate muscle ischemia, a defect associated with the loss of sarcolemmal nNOS in the dystrophic muscle fibres [12]. Sarcolemma-targeted nNOS attenuates α-adrenergic vasoconstriction in contracting muscles and improves muscle perfusion during exercise [47]. This process is defective in both *mdx* mice and patients with DMD [48, 9], thus promoting fatigue and injury of dystrophic muscles. Indeed, it has been recently demonstrated that naproxcinod at both 20 and 40 mg/kg is able to counteract skeletal muscle ischemia in *mdx* mice following one week of treatment, an effect not observed with naproxen [49]. Overall, these findings demonstrate an additional mechanism of action of naproxcinod, mediated by NO, which supports and explains the additional beneficial effects of naproxcinod over COX-mediated anti-inflammatory action.

However, it must also be added that the use of a mixed protocol, on both sedentary and exercised mice, as we did here, while providing valuable information may not provide a straightforward interpretation of the results as it would be with pure protocols based only on sedentary or exercised groups of mice. Thus, we cannot excluded other possible explanations of the results such as a different time-dependent effect of the two drugs, with naproxcinod being more effective than naproxen over chronic use.

Interestingly, naproxcinod showed an anti-inflammatory activity comparable to that of naproxen, reducing diaphragm inflammatory infiltrates, despite a bioavailability 3-fold lower than that of naproxen. These data are in agreement with previous pharmacokinetics studies where plasma availability of naproxen following naproxcinod oral administration was found to be lower (55 and 85 % in rats and mini-pigs, respectively) than that observed after administration of an equimolar dose of naproxen [50]. Similarly, in healthy volunteers relative plasma bioavailability of naproxen after naproxcinod administration was reported as 80–85 % compared with the availability of an equimolar dose of naproxen [51]. Despite these differences in availability of naproxen, no negative effects were observed on the efficacy of naproxcinod in reducing pain and inflammation in the hip and knee of patients with OA [34–36] in a way similar to that observed for the anti-inflammatory effects in *mdx* mice.

The data are explained by the additional anti-inflammatory properties associated with NO, which are not mediated by COX inhibition, namely the inhibition of both NF-kB activation and iNOS expression [52]. According to this, it has been shown that the precursor of NO, L-arginine, down-regulates levels of NF-κB in *mdx* muscles, resulting in lower activation of its downstream signaling and inhibition of two muscle-specific metalloproteinases, MMP-2 and MMP-9 [53]. Furthermore, long-term treatment with NO significantly reduces skeletal muscle inflammatory infiltrates and fibrosis by modulating the innate inflammatory response, increasing macrophage recruitment, and promoting a more efficient clearance of cell debris [54]. Both mechanisms of NF-κB inhibition and modulation of innate inflammatory response contribute to explain why the anti-inflammatory activity of naproxcinod is similar to that of naproxen in spite of its lower bioavailability. A further important finding, which may also explain the greater efficacy of naproxcinod on muscle function, is the reduction of fibrosis deposition in both diaphragm and heart. This effect could be ascribed to NO, since naproxen had no effect on this parameter. Consistent with this result, it has been recently demonstrated that NO regulates fibro-fatty tissue deposition in dystrophic skeletal muscle [45]. This effect may be mediated by the NO-dependent regulation of both miR-133a, a known regulator of collagen 1A1 expression [55], and miR-27b, a key inhibitor of adipocyte differentiation that controls the expression of peroxisome proliferator-activated receptor γ [56].

As well as relieving skeletal muscle disease, an effective therapy of DMD must also improve cardiac function, as heart failure is one of the key events leading to DMD patient decline and death [4]. The progression of DMD in cardiac muscle is much slower than in skeletal muscle and is characterized by reduced systolic function and cardiac arrhythmias [57, 58]. Unlike skeletal muscle, cardiac muscle is incapable of regeneration, since it lacks stem cells similar to the satellite cells of skeletal muscle [59]. In the present study, naproxcinod-treated animals showed a 35 % reduction in cardiac fibrosis compared with *mdx* control mice. This reduction is consistent with previous studies, where the inhibitory effects exerted by naproxcinod on cardiac fibrosis were also associated with a significant improvement of cardiac function [26], thus confirming the potential beneficial effects of naproxcinod treatment on DMD cardiomyopathy. In contrast, naproxen did not reduce cardiac fibrosis, suggesting once more that the effect on fibrosis can be ascribed to NO donation. In support of this, Wehling-Henricks et al. [60] found that overexpression of nNOS can effectively improve cardiomyopathy caused by dystrophin deficiency and that the improved cardiac function is associated with reduced cardiac fibrosis. Further evidence of the cardioprotective role of nNOS-derived NO has been recently demonstrated by the mitigation of heart pathology and improvement of heart function by nNOS gene therapy in aged *mdx* mice [61].

An aspect that has to be considered carefully when dealing with chronic therapies is the possibility of adverse events leading to significant tissue damage. In the case of NSAIDs a key issue is gastric damage [62]. Here, as expected, marked naproxen-induced gastric damage occurred after 6 months of treatment, while naproxcinod had a milder effect on the gastric mucosa. The safer gastric profile of naproxcinod could be explained by the known protective effects of NO in the gastrointestinal tract [63] but also by the reduced bioavailability of the compound.

## Conclusion

In conclusion, this study confirms the efficacy and the safety profile of naproxcinod in the *mdx* mouse model of DMD, and demonstrates a clear difference between naproxcinod and its parent drug naproxen. Naproxcinod has superior effects in improving muscle function and reducing both skeletal muscle and cardiac fibrosis compared to naproxen, with less gastro-intestinal side effects. In addition, naproxcinod could be the most appropriate CINOD for pediatric application considering that naproxen is approved for children and that drugs impacting on the pathway of NO such as tadalafil are currently under development for DMD patients.

Moreover, these data suggest that NO is responsible for the additional beneficial effects of naproxcinod over naproxen, suggesting a key role of NO donation in slowing disease progression in the *mdx* mouse model. Our findings conclusively indicate that naproxcinod has significant potential as a safe therapeutic option for the treatment of DMD.

## Additional files

Additional file 1:
**Naproxcinod administration does not alter body weight and food intake in** ***mdx*** **mice.** (a) Body weight and (b) food intake of *mdx* mice treated with vehicle (closed circles), naproxen at 20 mg/kg (grey triangles), or naproxcinod at 30 mg/kg (open squares) were monitored every week for 5 months. Data are presented as mean ± SEM. *N* = 8-10 mice/group. (TIFF 1270 kb) Additional file 2:
**Naproxcinod administration improves skeletal muscle force in sedentary** ***mdx*** **ice.** Skeletal muscle force assessed by WBT (WBT5 on the left and WBT10 on the right) following 1 (a and b), 2 (c and d) and 3 (e and f) months of treatment with either vehicle (black bar), 30 mg/kg naproxcinod (white bar) or 20 mg/kg naproxen (grey bar). Data are presented as mean ± SEM. *represents the comparison between vehicle and treatment groups. #represents the comparison versus naproxen-treated group. One-way ANOVA followed by Tukey post-hoc test. # *P* < 0.05, ****P* < 0.001. *N* = 8-10 mice/group. (TIFF 2337 kb) Additional file 3:
**Naproxcinod is safer than naproxen at gastric level.** Representative images of gastric sections from vehicle-treated *mdx* mice and *mdx* mice treated with either 30 mg/kg naproxcinod or 20 mg/kg naproxen. Bar = 100 μm. (TIFF 7053 kb)

## Abbreviations

ACN: Acetonitrile
BP: Blood pressure
CINODs: Cyclooxygenase (COX)-inhibiting NO donors
DGC: Dystrophin-glycoprotein complex
DMD: Duchenne muscular dystrophy
DMSO: Dimethyl sulfoxide
FPT: Forward pulling tension
H&E: Haematoxylin and eosin
ISDN: Isosorbide dinitrate
LC-MS/MS: Liquid chromatography-mass spectrometry
MMP-2 and MMP-9: Metalloproteinases 2 and 9
NF-κB: Nuclear factor kappa-light-chain-enhancer of activated B cells
nNOS: Neuronal nitric oxide synthase
NO: Nitric oxide
NS: Not significant
NSAID: Non steroidal anti-inflammatory drug
SIRT1: Silent mating type information regulation 2 homolog
SOP: Standard operating procedures
OA: Osteoarthritis
WBT: Whole body tension
