# Supplementary figures and images for: Naproxcinod shows significant advantages over naproxen in the mdx model of Duchenne Muscular Dystrophy
Source: Orphanet J Rare Dis. 2015 Aug 22;10:101. doi: 10.1186/s13023-015-0311-0 (PMC4546261; doi:10.1186/s13023-015-0311-0)

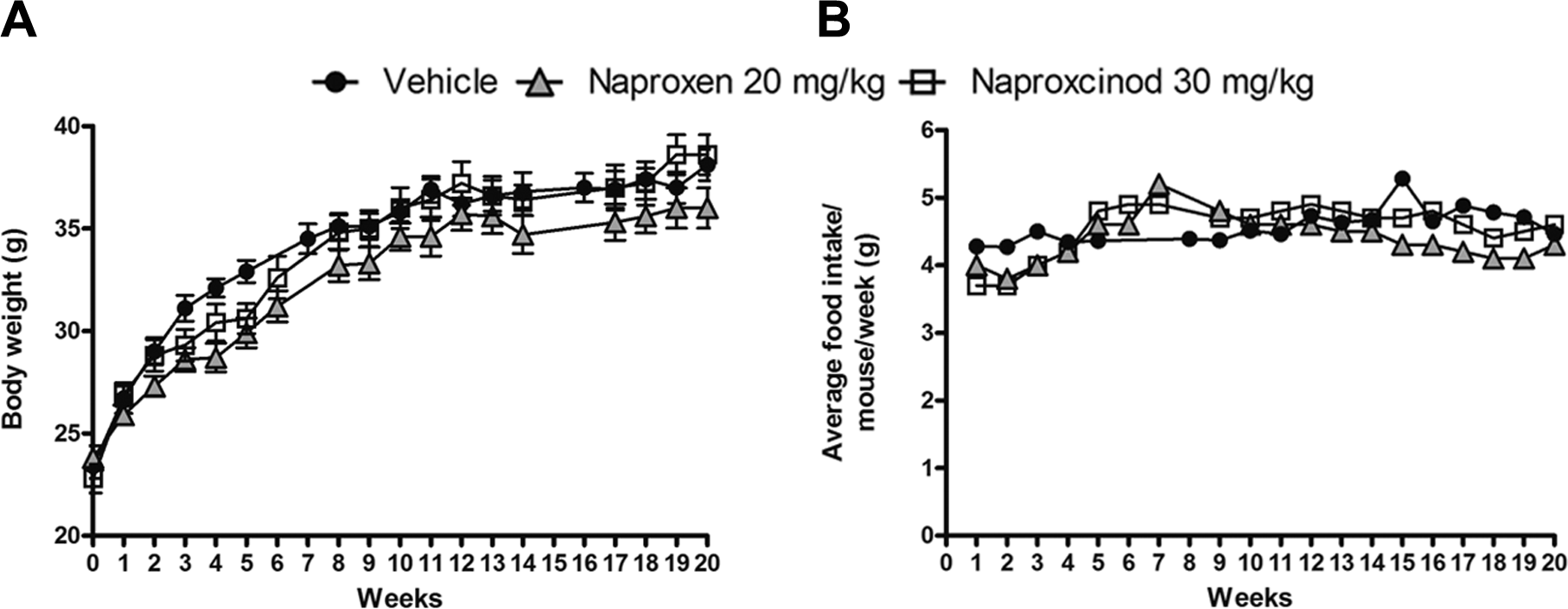

Supplement: Additional file 1: — Naproxcinod administration does not alter body weight and food intake in mdx mice. (a) Body weight and (b) food intake of mdx mice treated with vehicle (closed circles), naproxen at 20 mg/kg (grey triangles), or naproxcinod at 30 mg/kg (open squares) were monitored every week for 5 months. Data are presented as mean ± SEM. N = 8-10 mice/group. (TIFF 1270 kb) [file 13023_2015_311_MOESM1_ESM.tif]

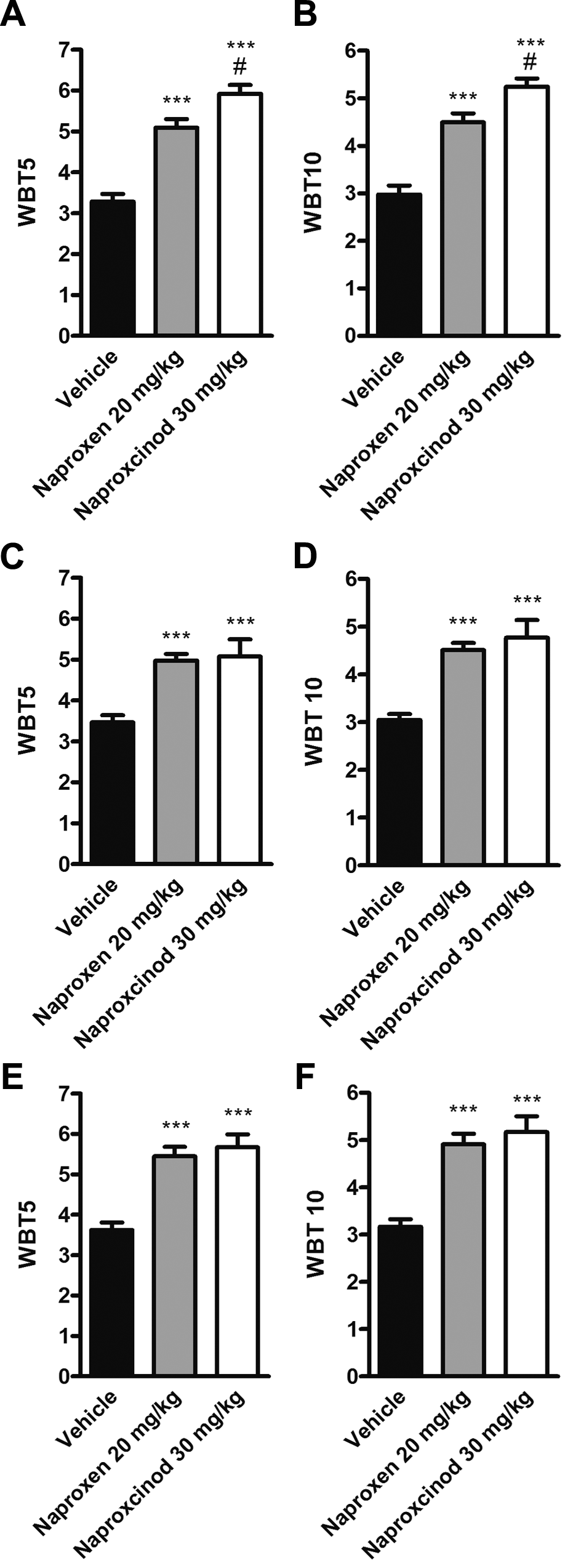

Supplement: Additional file 2: — Naproxcinod administration improves skeletal muscle force in sedentary mdx ice. Skeletal muscle force assessed by WBT (WBT5 on the left and WBT10 on the right) following 1 (a and b), 2 (c and d) and 3 (e and f) months of treatment with either vehicle (black bar), 30 mg/kg naproxcinod (white bar) or 20 mg/kg naproxen (grey bar). Data are presented as mean ± SEM. *represents the comparison between vehicle and treatment groups. #represents the comparison versus naproxen-treated group. One-way ANOVA followed by Tukey post-hoc test. # P < 0.05, ***P < 0.001. N = 8-10 mice/group. (TIFF 2337 kb) [file 13023_2015_311_MOESM2_ESM.tif]

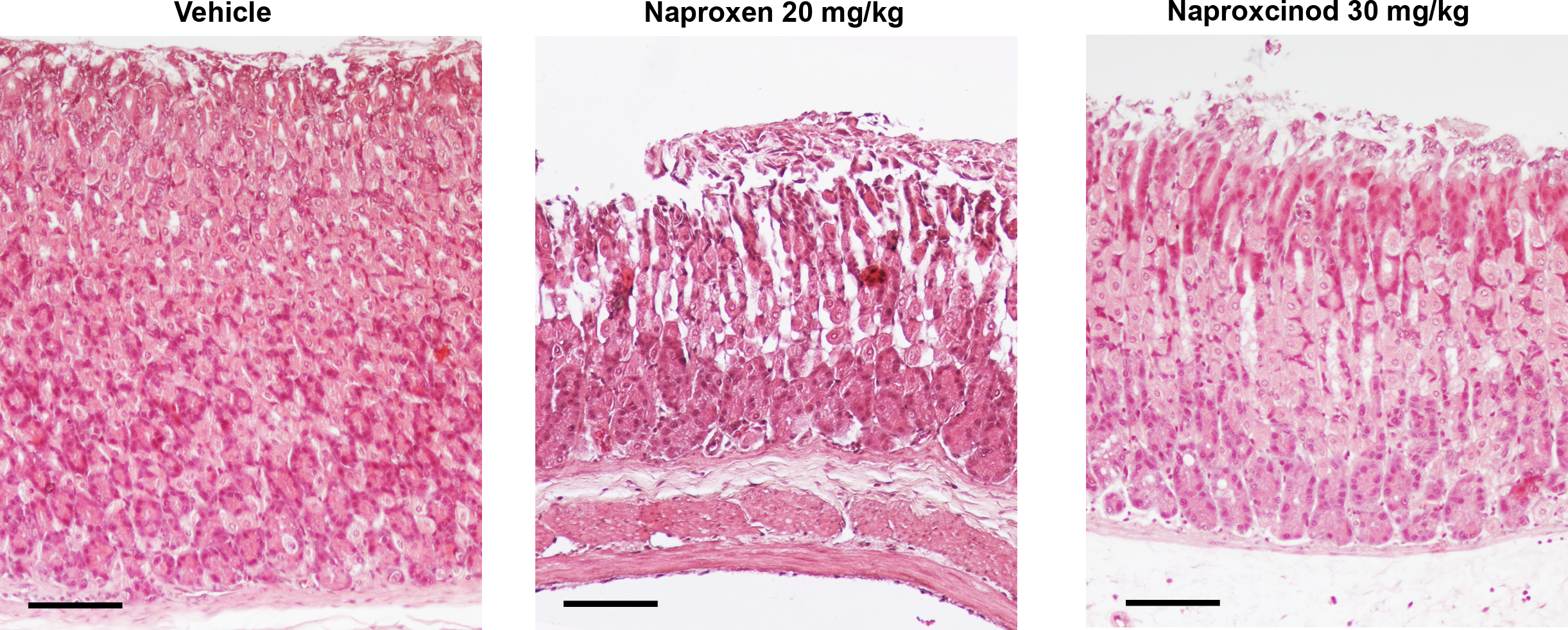

Supplement: Additional file 3: — Naproxcinod is safer than naproxen at gastric level. Representative images of gastric sections from vehicle-treated mdx mice and mdx mice treated with either 30 mg/kg naproxcinod or 20 mg/kg naproxen. Bar = 100 μm. (TIFF 7053 kb) [file 13023_2015_311_MOESM3_ESM.tif]
